# Machine learning in project analytics: a data-driven framework and case study

**DOI:** 10.1038/s41598-022-19728-x

**Published:** 2022-09-09

**Authors:** Shahadat Uddin, Stephen Ong, Haohui Lu

**Affiliations:** School of Project Management, The University of Sydney, Level 2, 21 Ross St, Forest Lodge, NSW 2037 Australia

**Keywords:** Applied mathematics, Computational science

## Abstract

The analytic procedures incorporated to facilitate the delivery of projects are often referred to as project analytics. Existing techniques focus on retrospective reporting and understanding the underlying relationships to make informed decisions. Although machine learning algorithms have been widely used in addressing problems within various contexts (e.g., streamlining the design of construction projects), limited studies have evaluated pre-existing machine learning methods within the delivery of construction projects. Due to this, the current research aims to contribute further to this convergence between artificial intelligence and the execution construction project through the evaluation of a specific set of machine learning algorithms. This study proposes a machine learning-based data-driven research framework for addressing problems related to project analytics. It then illustrates an example of the application of this framework. In this illustration, existing data from an open-source data repository on construction projects and cost overrun frequencies was studied in which several machine learning models (Python’s Scikit-learn package) were tested and evaluated. The data consisted of 44 independent variables (from materials to labour and contracting) and one dependent variable (project cost overrun frequency), which has been categorised for processing under several machine learning models. These models include support vector machine, logistic regression, *k*-nearest neighbour, random forest, stacking (ensemble) model and artificial neural network. Feature selection and evaluation methods, including the Univariate feature selection, Recursive feature elimination, SelectFromModel and confusion matrix, were applied to determine the most accurate prediction model. This study also discusses the generalisability of using the proposed research framework in other research contexts within the field of project management. The proposed framework, its illustration in the context of construction projects and its potential to be adopted in different contexts will significantly contribute to project practitioners, stakeholders and academics in addressing many project-related issues.

## Introduction

Successful projects require the presence of appropriate information and technology^1^. Project analytics provides an avenue for informed decisions to be made through the lifecycle of a project. Project analytics applies various statistics (e.g., earned value analysis or Monte Carlo simulation) among other models to make evidence-based decisions. They are used to manage risks as well as project execution^2^. There is a tendency for project analytics to be employed due to other additional benefits, including an ability to forecast and make predictions, benchmark with other projects, and determine trends such as those that are time-dependent^3–5^. There has been increasing interest in project analytics and how current technology applications can be incorporated and utilised^6^. Broadly, project analytics can be understood on five levels^4^. The first is *descriptive analytics* which incorporates retrospective reporting. The second is known as *diagnostic analytics*, which aims to understand the interrelationships and underlying causes and effects. The third is *predictive analytics* which seeks to make predictions. Subsequent to this is *prescriptive analytics*, which prescribes steps following predictions. Finally, *cognitive analytics* aims to predict future problems. The first three levels can be applied with ease with the help of technology. The fourth and fifth steps require data that is generally more difficult to obtain as they may be less accessible or unstructured. Further, although project key performance indicators can be challenging to define^2^, identifying common measurable features facilitates this^7^. It is anticipated that project analytics will continue to experience development due to its direct benefits to the major baseline measures focused on productivity, profitability, cost, and time^8^. The nature of project management itself is fluid and flexible, and project analytics allows an avenue for which machine learning algorithms can be applied^9^.

Machine learning within the field of project analytics falls into the category of cognitive analytics, which deals with problem prediction. Generally, machine learning explores the possibilities of computers to improve processes through training or experience^10^. It can also build on the pre-existing capabilities and techniques prevalent within management to accomplish complex tasks^11^. Due to its practical use and broad applicability, recent developments have led to the invention and introduction of newer and more innovative machine learning algorithms and techniques. Artificial intelligence, for instance, allows for software to develop computer vision, speech recognition, natural language processing, robot control, and other applications^10^. Specific to the construction industry, it is now used to monitor construction environments through a virtual reality and building information modelling replication^12^ or risk prediction^13^. Within other industries, such as consumer services and transport, machine learning is being applied to improve consumer experiences and satisfaction^10,14^ and reduce the human errors of traffic controllers^15^. Recent applications and development of machine learning broadly fall into the categories of classification, regression, ranking, clustering, dimensionality reduction and manifold learning^16^. Current learning models include linear predictors, boosting, stochastic gradient descent, kernel methods, and nearest neighbour, among others^11^. Newer and more applications and learning models are continuously being introduced to improve accessibility and effectiveness.

Specific to the management of construction projects, other studies have also been made to understand how copious amounts of project data can be used^17^, the importance of ontology and semantics throughout the nexus between artificial intelligence and construction projects^18,19^ as well as novel approaches to the challenges within this integration of fields^20–22^. There have been limited applications of pre-existing machine learning models on construction cost overruns. They have predominantly focussed on applications to streamline the design processes within construction^23–26^, and those which have investigated project profitability have not incorporated the types and combinations of algorithms used within this study^6,27^. Furthermore, existing applications have largely been skewed towards one type or another^28,29^.

**Figure 1:**
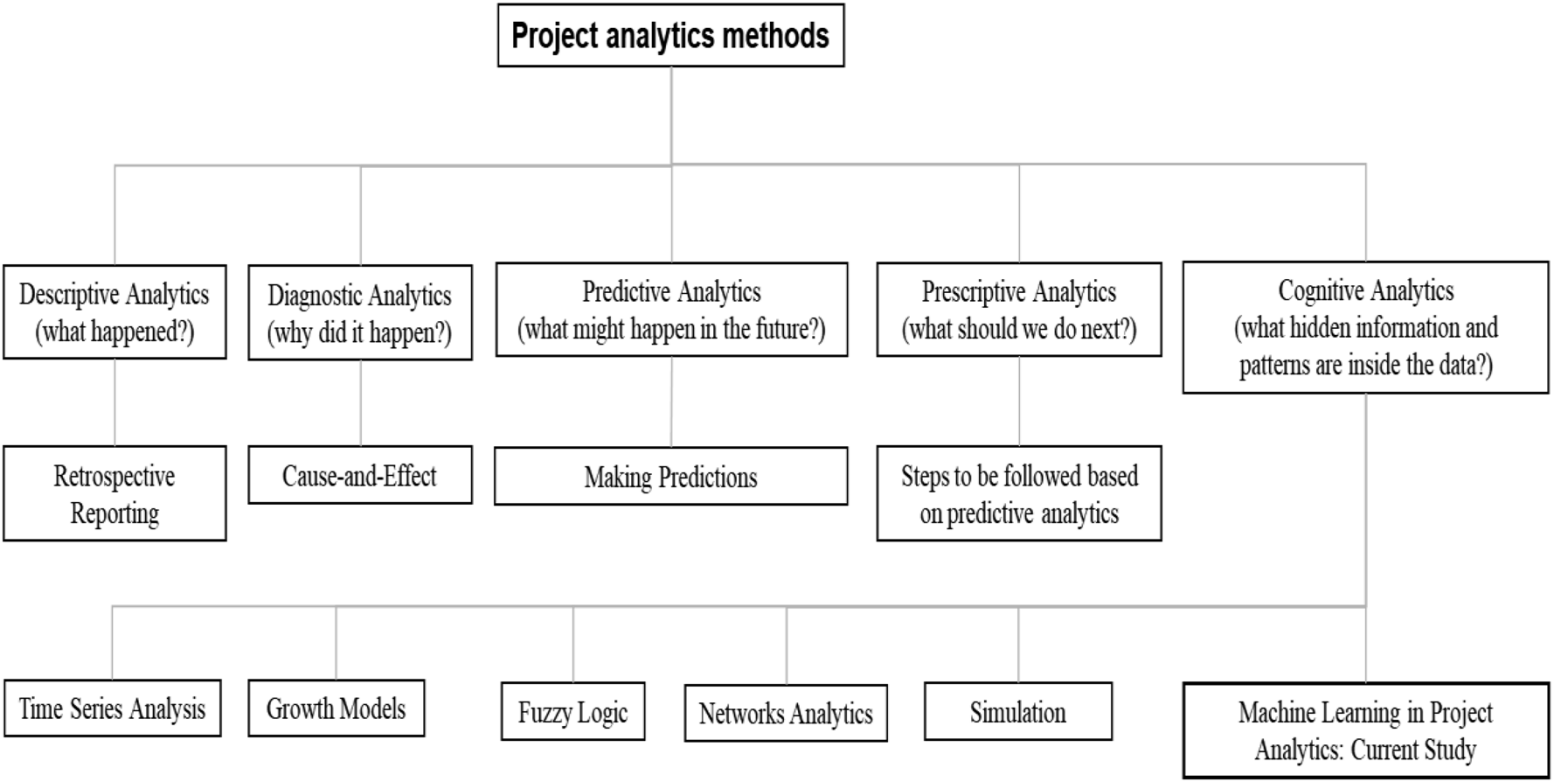
A tree diagram of different project analytics methods. It also shows where the current study belongs to. Although earned value analysis is commonly used in project analytics, we do not include it in this figure since it is used in the first three levels of project analytics.

In addition to the frequently used earned value method (EVM), researchers have been applying many other powerful quantitative methods to address a diverse range of project analytics research problems over time. Examples of those methods include time series analysis, fuzzy logic, simulation, network analytics, and network correlation and regression. Time series analysis uses longitudinal data to forecast an underlying project's future needs, such as the time and cost^30–32^. Few other methods are combined with EVM to find a better solution for the underlying research problems. For example, Narbaev and De Marco^33^ integrated growth models and EVM for forecasting project cost at completion using data from construction projects. For analysing the ongoing progress of projects having ambiguous or linguistic outcomes, fuzzy logic is often combined with EVM^34–36^. Yu et al.^36^ applied fuzzy theory and EVM for schedule management. Ponz-Tienda et al.^35^ found that using fuzzy arithmetic on EVM provided more objective results in uncertain environments than the traditional methodology. Bonato et al.^37^ integrated EVM with Monte Carlo simulation to predict the final cost of three engineering projects. Batselier and Vanhoucke^38^ compared the accuracy of the project time and cost forecasting using EVM and simulation. They found that the simulation results supported findings from the EVM. Network methods are primarily used to analyse project stakeholder networks. Yang and Zou^39^ developed a social network theory-based model to explore stakeholder-associated risks and their interactions in complex green building projects. Uddin^40^ proposed a social network analytics-based framework for analysing stakeholder networks. Ong and Uddin^41^ further applied network correlation and regression to examine the co-evolution of stakeholder networks in collaborative healthcare projects. Although many other methods have already been used, as evident in the current literature, machine learning methods or models are yet to be adopted for addressing research problems related to project analytics. The current investigation is derived from the cognitive analytics component of project analytics. It proposes an approach for determining hidden information and patterns to assist with project delivery. Figure 1 illustrates a tree diagram showing different levels of project analytics and their associated methods from the literature. It also illustrates existing methods within the cognitive component of project analytics to where the application of machine learning is situated contextually.

Machine learning models have several notable advantages over traditional statistical methods that play a significant role in project analytics^42^. First, machine learning algorithms can quickly identify trends and patterns by simultaneously analysing a large volume of data. Second, they are more capable of continuous improvement. Machine learning algorithms can improve their accuracy and efficiency for decision-making through subsequent training from potential new data. Third, machine learning algorithms efficiently handle multi-dimensional and multi-variety data in dynamic or uncertain environments. Fourth, they are compelling to automate various decision-making tasks. For example, machine learning-based sentiment analysis can easily a negative tweet and can automatically take further necessary steps. Last but not least, machine learning has been helpful across various industries, for example, defence to education^43^. Current research has seen the development of several different branches of artificial intelligence (including robotics, automated planning and scheduling and optimisation) within safety monitoring, risk prediction, cost estimation and so on^44^. This has progressed from the applications of regression on project cost overruns^45^ to the current deep-learning implementations within the construction industry^46^. Despite this, the uses remain largely limited and are still in a developmental state. The benefits of applications are noted, such as optimising and streamlining existing processes; however, high initial costs form a barrier to accessibility^44^.

The primary goal of this study is to demonstrate the applicability of different machine learning algorithms in addressing problems related to project analytics. Limitations in applying machine learning algorithms within the context of construction projects have been explored previously. However, preceding research has mainly been conducted to improve the design processes specific to construction^23,24^, and those investigating project profitabilities have not incorporated the types and combinations of algorithms used within this study^6,27^. For instance, preceding research has incorporated a different combination of machine-learning algorithms in research of predicting construction delays^47^. This study first proposed a machine learning-based data-driven research framework for project analytics to contribute to the proposed study direction. It then applied this framework to a case study of construction projects. Although there are three different machine learning algorithms (supervised, unsupervised and semi-supervised), the supervised machine learning models are most commonly used due to their efficiency and effectiveness in addressing many real-world problems^48^. Therefore, we will use *machine learning* to represent *supervised machine learning* throughout the rest of this article. The contribution of this study is significant in that it considers the applications of machine learning within project management. Project management is often thought of as being very fluid in nature, and because of this, applications of machine learning are often more difficult^9,49^. Further to this, existing implementations have largely been limited to safety monitoring, risk prediction, cost estimation and so on^44^. Through the evaluation of machine-learning applications, this study further demonstrates a case study for which algorithms can be used to consider and model the relationship between project attributes and a project performance measure (i.e., cost overrun frequency).

## Machine learning-based framework for project analytics

### When and why machine learning for project analytics?

Machine learning models are typically used for research problems that involve predicting the classification outcome of a categorical dependent variable. Therefore, they can be applied in the context of project analytics if the underlying objective variable is a categorical one. If that objective variable is non-categorical, it must first be converted into a categorical variable. For example, if the objective or target variable is the project cost, we can convert this variable into a categorical variable by taking only two possible values. The first value would be 0 to indicate a low-cost project, and the second could be 1 for showing a high-cost project. The average or median cost value for all projects under consideration can be considered for splitting project costs into low-cost and high-cost categories.

**Figure 2:**
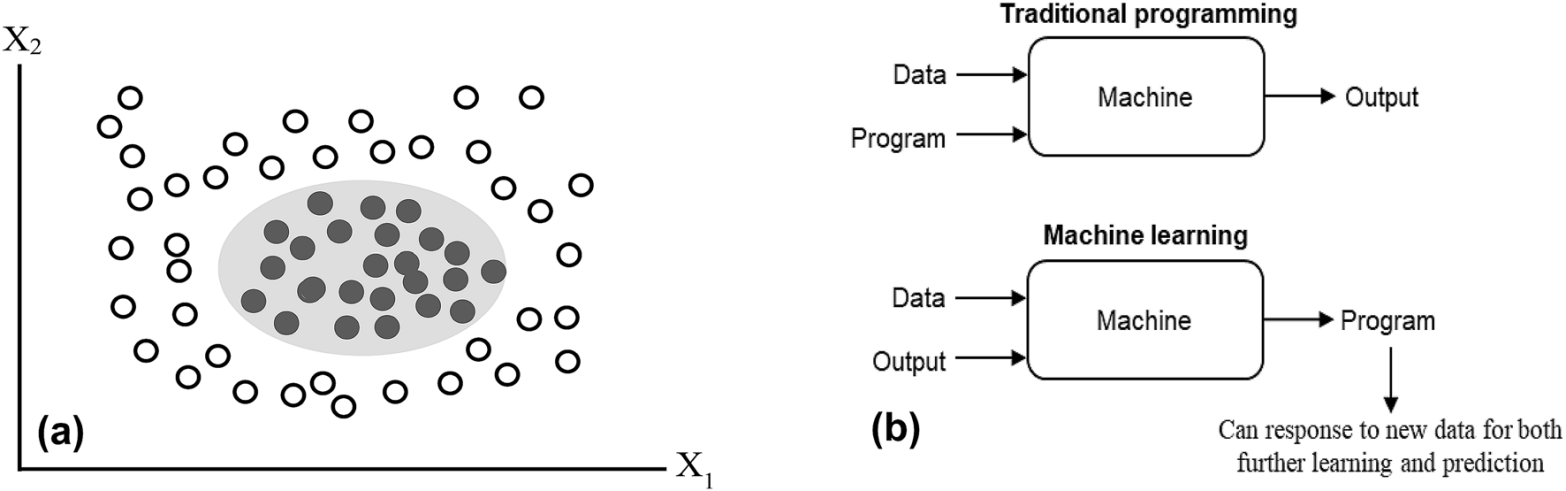
(**a**) An illustration showing the superior performance of machine learning models compared with the traditional statistical models using an abstract dataset with two attributes (X_1_ and X_2_). The data points within this abstract dataset consist of two classes: one represented with a transparent circle and the second class illustrated with a black-filled circle. These data points are non-linear but separable. Traditional statistical models (e.g., ordinary least square regression) will not accurately separate these data points. However, any machine learning model can easily separate them without making errors; and (**b**) Traditional programming versus machine learning.

For data-driven decision-making, machine learning models are advantageous. This is because traditional statistical methods (e.g., ordinary least square (OLS) regression) make assumptions about the underlying research data to produce explicit formulae for the objective target measures. Unlike these statistical methods, machine learning algorithms figure out patterns on their own directly from the data. For instance, for a non-linear but separable dataset, an OLS regression model will not be the right choice due to its assumption that the underlying data must be linear. However, a machine learning model can easily separate the dataset into the underlying classes. Figure 2(a) presents a situation where machine learning models perform better than traditional statistical methods.

Similarly, machine learning models are compelling if the underlying research dataset has many attributes or independent measures. Such models can identify features that significantly contribute to the corresponding classification performance regardless of their distributions or collinearity. Traditional statistical methods have become prone to biased results when there exists a correlation between independent variables. Machine learning-based current studies specific to project analytics have been largely limited. Despite this, there have been tangential studies on the use of artificial intelligence to improve cost estimations as well as risk prediction^44^. Additionally, models have been implemented in the optimisation of existing processes^50^.

### Machine learning versus traditional programming

Machine learning can be thought of as a process of teaching a machine (i.e., computers) to learn from data and adjust or apply its present knowledge when exposed to new data^42^. It is a type of artificial intelligence that enables computers to learn from examples or experiences. Traditional programming requires some input data and some logic in the form of code (program) to generate the output. Unlike traditional programming, the input data and their corresponding output are fed to an algorithm to create a program in machine learning. This resultant program can capture powerful insights into the data pattern and can be used to predict future outcomes. Figure 2(b) shows the difference between machine learning and traditional programming.

### Proposed machine learning-based framework

**Figure 3:**
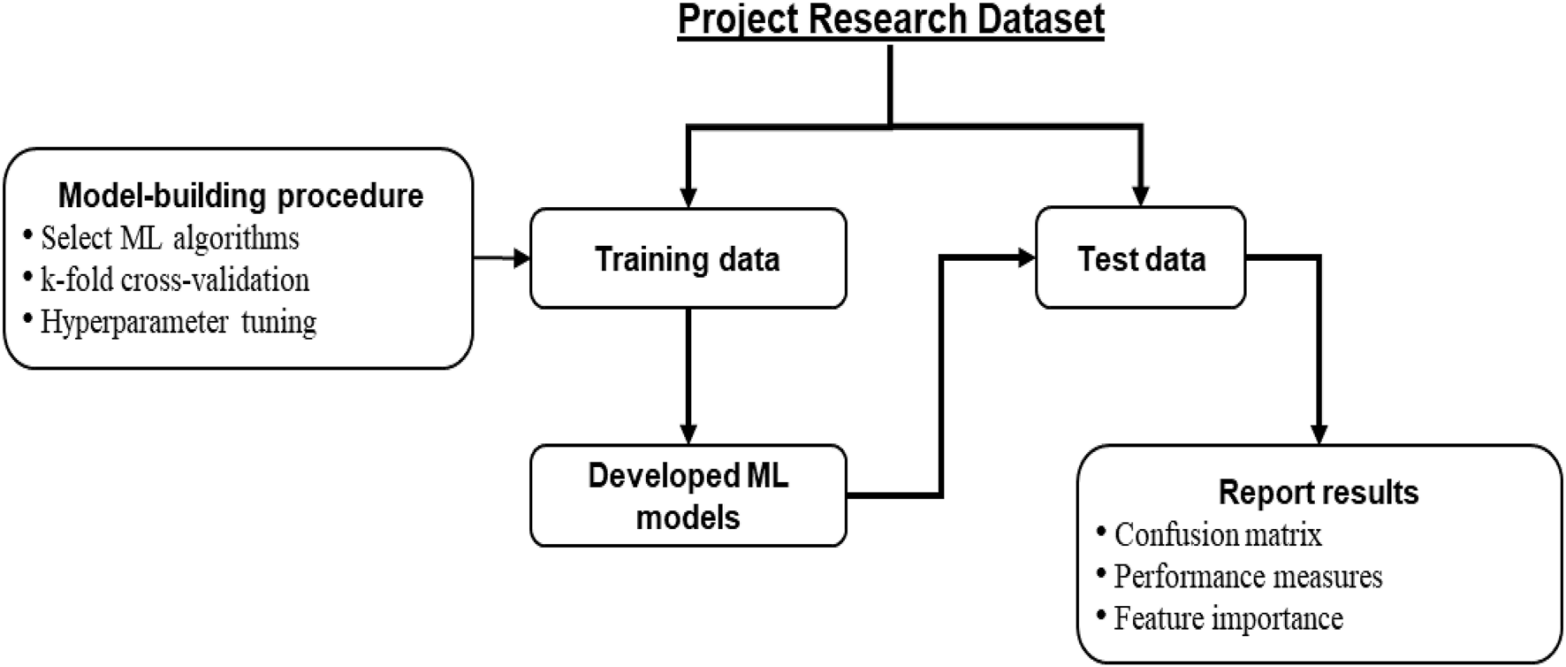
The proposed machine learning-based data-driven framework.

Figure 3 illustrates the proposed machine learning-based research framework of this study. The framework starts with breaking the project research dataset into the training and test components. As mentioned in the previous section, the research dataset may have many categorical and/or nominal independent variables, but its single dependent variable must be categorical. Although there is no strict rule for this split, the training data size is generally more than or equal to 50% of the original dataset^48^.

Machine learning algorithms can handle variables that have only numerical outcomes. So, when one or more of the underlying categorical variables have a textual or string outcome, we must first convert them into the corresponding numerical values. Suppose a variable can take only three textual outcomes (low, medium and high). In that case, we could consider, for example, 1 to represent *low*, 2 to represent *medium*, and 3 to represent *high*. Other statistical techniques, such as the RIDIT (relative to an identified distribution) scoring^51^, can also be used to convert ordered categorical measurements into quantitative ones. RIDIT is a parametric approach that uses probabilistic comparison to determine the statistical differences between ordered categorical groups. The remaining components of the proposed framework have been briefly described in the following subsections.

#### Model-building procedure

The next step of the framework is to follow the model-building procedure to develop the desired machine learning models using the training data. The first step of this procedure is to select suitable machine learning algorithms or models. Among the available machine learning algorithms, the commonly used ones are support vector machine, logistic regression, *k*-nearest neighbours, artificial neural network, decision tree and random forest^52^. One can also select an ensemble machine learning model as the desired algorithm. An ensemble machine learning method uses multiple algorithms or the same algorithm multiple times to achieve better predictive performance than could be obtained from any of the constituent learning models alone^52^. Three widely used ensemble approaches are bagging, boosting and stacking. In bagging, the research dataset is divided into different equal-sized subsets. The underlying machine learning algorithm is then applied to these subsets for classification. In boosting, a random sample of the dataset is selected and then fitted and trained sequentially with different models to compensate for the weakness observed in the immediately used model. Stacking combined different weak machine learning models in a heterogeneous way to improve the predictive performance. For example, the random forest algorithm is an ensemble of different decision tree models^42^.

Second, each selected machine learning model will be processed through the *k*-fold cross-validation approach to improve predictive efficiency. In *k*-fold cross-validation, the training data is divided into *k* folds. In an iteration, the *(k-1)* folds are used to train the selected machine models, and the remaining last fold isF used for validation purposes. This iteration process continues until each *k* folds will get a turn to be used for validation purposes. The final predictive efficiency of the trained models is based on the average values from the outcomes of these iterations. In addition to this average value, researchers use the standard deviation of the results from different iterations as the predictive training efficiency. Supplementary Fig 1 shows an illustration of the *k*-fold cross-validation.

Third, most machine learning algorithms require a pre-defined value for their different parameters, known as hyperparameter tuning. The settings of these parameters play a vital role in the achieved performance of the underlying algorithm. For a given machine learning algorithm, the optimal value for these parameters can be different from one dataset to another. The same algorithm needs to run multiple times with different parameter values to find its optimal parameter value for a given dataset. Many algorithms are available in the literature, such as the Grid search^53^, to find the optimal parameter value. In the Grid search, hyperparameters are divided into discrete grids. Each grid point represents a specific combination of the underlying model parameters. The parameter values of the point that results in the best performance are the optimal parameter values^53^.

#### Testing of the developed models and reporting results

Once the desired machine learning models have been developed using the training data, they need to be tested using the test data. The underlying trained model is then applied to predict its dependent variable for each data instance. Therefore, for each data instance, two categorical outcomes will be available for its dependent variable: one predicted using the underlying trained model, and the other is the actual category. These predicted and actual categorical outcome values are used to report the results of the underlying machine learning model.

The fundamental tool to report results from machine learning models is the confusion matrix, which consists of four integer values^48^. The first value represents the number of positive cases correctly identified as positive by the underlying trained model (true-positive). The second value indicates the number of positive instances incorrectly identified as negative (false-negative). The third value represents the number of negative cases incorrectly identified as positive (false-positive). Finally, the fourth value indicates the number of negative instances correctly identified as negative (true-negative). Researchers also use a few performance measures based on the four values of the confusion matrix to report machine learning results. The most used measure is accuracy which is the ratio of the number of correct predictions (true-positive + true-negative) and the total number of data instances (sum of all four values of the confusion matrix). Other measures commonly used to report machine learning results are precision, recall and F1-score. Precision refers to the ratio between true-positives and the total number of positive predictions (i.e., true-positive + false-positive), often used to indicate the quality of a positive prediction made by a model^48^. Recall, also known as the true-positive rate, is calculated by dividing true-positive by the number of data instances that should have been predicted as positive (i.e., true-positive + false-negative). F1-score is the harmonic mean of the last two measures, i.e., [(2 × Precision × Recall)/(Precision + Recall)] and the error-rate equals to (1-Accuracy).

Another essential tool for reporting machine learning results is variable or feature importance, which identifies a list of independent variables (features) contributing most to the classification performance. The importance of a variable refers to how much a given machine learning algorithm uses that variable in making accurate predictions^54^. The widely used technique for identifying variable importance is the principal component analysis. It reduces the dimensionality of the data while minimising information loss, which eventually increases the interpretability of the underlying machine learning outcome. It further helps in finding the important features in a dataset as well as plotting them in 2D and 3D^54^.

### Ethical approval

Ethical approval is not required for this study since this study used publicly available data for research investigation purposes. All research was performed in accordance with relevant guidelines/regulations.

### Informed consent

Due to the nature of the data sources, informed consent was not required for this study.

## Case study: an application of the proposed framework

This section illustrates an application of this study’s proposed framework (Fig. 2) in a construction project context. We will apply this framework in classifying projects into two classes based on their cost overrun experience. Projects *rarely* experience a delay belonging to the first class (Rare class). The second class indicates those projects that *often* experience a delay (Often class). In doing so, we consider a list of independent variables or features.

### Data source

The research dataset is taken from an open-source data repository, Kaggle^55^. This survey-based research dataset was collected to explore the causes of the project cost overrun in Indian construction projects^45^, consisting of 44 independent variables or features and one dependent variable. The independent variables cover a wide range of cost overrun factors, from materials and labour to contractual issues and the scope of the work. The dependent variable is the frequency of experiencing project cost overrun (rare or often). The dataset size is 139; 65 belong to the *rare* class, and the remaining 74 are from the *often* class. We converted each categorical variable with a textual or string outcome into an appropriate numerical value range to prepare the dataset for machine learning analysis. For example, we used 1 and 2 to represent *rare* and *often* class, respectively. The correlation matrix among the 44 features is presented in Supplementary Fig 2.

### Machine learning algorithms

This study considered four machine learning algorithms to explore the causes of project cost overrun using the research dataset mentioned above. They are support vector machine, logistic regression, *k-*nearest neighbours and random forest.

Support vector machine (SVM) is a process applied to understand data. For instance, if one wants to determine and interpret which projects are classified as programmatically successful through the processing of precedent data information, SVM would provide a practical approach for prediction. SVM functions by assigning labels to objects^56^. The comparison attributes are used to cluster these objects into different groups or classes by maximising their marginal distances and minimising the classification errors. The attributes are plotted multi-dimensionally, allowing a separation line, known as a *hyperplane*, see supplementary Fig 3(a), to distinguish between underlying classes or groups^52^. Support vectors are the data points that lie closest to the decision boundary on both sides. In Supplementary Fig 3(a), they are the circles (both transparent and shaded ones) close to the hyperplane. Support vectors play an essential role in deciding the position and orientation of the hyperplane. Various computational methods, including a kernel function to create more derived attributes, are applied to accommodate this process^56^. Support vector machines are not only limited to binary classes but can also be generalised to a larger variety of classifications. This is accomplished through the training of separate SVMs^56^.

Logistic regression (LR) builds on the linear regression model and predicts the outcome of a dichotomous variable^57^; for example, the presence or absence of an event. It uses a scatterplot to understand the connection between an independent variable and one or more dependent variables (see Supplementary Fig 3(b)). LR model fits the data to a sigmoidal curve instead of fitting it to a straight line. The natural logarithm is considered when developing the model. It provides a value between 0 and 1 that is interpreted as the probability of class membership. Best estimates are determined by developing from approximate estimates until a level of stability is reached^58^. Generally, LR offers a straightforward approach for determining and observing interrelationships. It is more efficient compared to ordinary regressions^59^.

*k*-nearest neighbours (KNN) algorithm uses a process that plots prior information and applies a specific sample size (*k*) to the plot to determine the most likely scenario^52^. This method finds the nearest training examples using a distance measure. The final classification is made by counting the most common scenario or *votes* present within the specified sample. As illustrated in Supplementary Fig 3(c), the closest four nearest neighbours in the small circle are three grey squares and one white square. The majority class is grey. Hence, KNN will predict the instance (i.e., ***Χ***) as grey. On the other hand, if we look at the larger circle of the same figure, the nearest neighbours consist of ten white squares and four grey squares. The majority class is white. Thus, KNN will classify the instance as white. KNN’s advantage lies in its ability to produce a simplified result and handle missing data^60^. In summary, KNN utilises similarities (as well as differences) and distances in the process of developing models.

Random forest (RF) is a machine learning process that consists of many decision trees. A decision tree is a tree-like structure where each internal node represents a test on the input attribute. It may have multiple internal nodes at different levels, and the leaf or terminal nodes represent the decision outcomes. It produces a classification outcome for a distinctive and separate part to the input vector. For non-numerical processes, it considers the average value, and for discrete processes, it considers the number of *votes*^52^. Supplementary Fig 3(d) shows three decision trees to illustrate the function of a random forest. The outcomes from trees 1, 2 and 3 are class B, class A and class A, respectively. According to the majority vote, the final prediction will be class A. Because it considers specific attributes, it can have a tendency to emphasise specific attributes over others, which may result in some attributes being unevenly weighted^52^. Advantages of the random forest include its ability to handle multidimensionality and multicollinearity in data despite its sensitivity to sampling design.

Artificial neural network (ANN) simulates the way in which human brains work. This is accomplished by modelling logical propositions and incorporating weighted inputs, a transfer and one output^61^ (Supplementary Fig 3(e)). It is advantageous because it can be used to model non-linear relationships and handle multivariate data^62^. ANN learns through three major avenues. These include error-back propagation (supervised), the Kohonen (unsupervised) and the counter-propagation ANN (supervised)^62^. There are two types of ANN—supervised and unsupervised. ANN has been used in a myriad of applications ranging from pharmaceuticals^61^ to electronic devices^63^. It also possesses great levels of fault tolerance^64^ and learns by example and through self-organisation^65^.

Ensemble techniques are a type of machine learning methodology in which numerous basic classifiers are combined to generate an optimal model^66^. An ensemble technique considers many models and combines them to form a single model, and the final model will eliminate the weaknesses of each individual learner, resulting in a powerful model that will improve model performance. The stacking model is a general architecture comprised of two classifier levels: base classifier and meta-learner^67^. The base classifiers are trained with the training dataset, and a new dataset is constructed for the meta-learner. Afterwards, this new dataset is used to train the meta-classifier. This study uses four models (SVM, LR, KNN and RF) as base classifiers and LR as a meta learner, as illustrated in Supplementary Fig 3(f).

### Feature selection

The process of selecting the optimal feature subset that significantly influences the predicted outcomes, which may be efficient to increase model performance and save running time, is known as feature selection. This study considers three different feature selection approaches. They are the Univariate feature selection (UFS), Recursive feature elimination (RFE) and SelectFromModel (SFM) approach. UFS examines each feature separately to determine the strength of its relationship with the response variable^68^. This method is straightforward to use and comprehend and helps acquire a deeper understanding of data. In this study, we calculate the chi-square values between features. RFE is a type of backwards feature elimination in which the model is fit first using all features in the given dataset and then removing the least important features one by one^69^. After that, the model is refit until the desired number of features is left over, which is determined by the parameter. SFM is used to choose effective features based on the feature importance of the best-performing model^70^. This approach selects features by establishing a threshold based on feature significance as indicated by the model on the training set. Those characteristics whose feature importance is more than the threshold are chosen, while those whose feature importance is less than the threshold are deleted. In this study, we apply SFM after we compare the performance of four machine learning methods. Afterwards, we train the best-performing model again using the features from the SFM approach.

### Findings from the case study

We split the dataset into 70:30 for training and test purposes of the four selected machine learning algorithms. We used Python’s Scikit-learn package for implementing these algorithms^70^. Using the training data, we first developed six models based on these six algorithms. We used fivefold validation and target to improve the accuracy value. Then, we applied these models to the test data. We also executed all required hyperparameter tunings for each algorithm for the possible best classification outcome. Table 1 shows the performance outcomes for each algorithm during the training and test phase. The hyperparameter settings for each algorithm have been listed in Supplementary Table 1.

**Table 1:** The performance of the six machine learning algorithms for the case study.

(a) Training phase (values are in %)					
Machine learning algorithm	Training accuracy (standard deviation)				
Support vector machine	69.89 (9.09)				
Logistic regression	68.26 (9.39)				
*k*-nearest neighbours	76.98 (8.27)				
Random forest	78.14 (8.92)				
Stacking (ensemble) model	74.05 (9.56)				
Artificial neural network	67.50 (3.54)				

(b) Testing phase (values are in %)					
Machine learning algorithm	Accuracy	Precision	Recall	F1-Score	Error-rate
Support vector machine	72.50	65.00	76.47	70.27	27.50
Logistic regression	67.50	60.00	70.59	64.86	32.50
*k*-nearest neighbours	72.50	65.00	76.47	70.27	27.50
Random forest	77.50	68.18	88.24	76.92	22.50
Stacking (ensemble) model	70.00	63.16	70.59	66.67	30.00
Artificial neural network	72.50	65.00	76.47	70.27	27.50

As revealed in Table 1, random forest outperformed the other three algorithms in terms of accuracy for both the training and test phases. It showed an accuracy of 78.14% and 77.50% for the training and test phases, respectively. The second-best performer in the training phase is *k-*nearest neighbours (76.98%), and for the test phase, it is the support vector machine, *k-*nearest neighbours and artificial neural network (72.50%).

Since random forest showed the best performance, we explored further based on this algorithm. We applied the three approaches (UFS, RFE and SFM) for feature optimisation on the random forest. The result is presented in Table 2. SFM shows the best outcome among these three approaches. Its accuracy is 85.00%, whereas the accuracies of USF and RFE are 77.50% and 72.50%, respectively. As can be seen in Table 2, the accuracy for the testing phase increases from 77.50% in Table 1(b) to 85.00% with the SFM feature optimisation. Table 3 shows the 19 selected features from the SFM output. Out of 44 features, SFM found that 19 of them play a significant role in predicting the outcomes.

**Table 2:** The performance of the random forest algorithm from the testing phase using three different attribute/feature optimisation approaches. Values are in percentage.

Feature optimisation approach	Accuracy	Precision	Recall	F1-score	Error-rate
Random forest with features from UFS	77.50	66.67	94.12	78.05	22.50
Random forest with features from REF	72.50	63.64	82.35	71.19	27.50
Random forest with features from SFM	85.00	76.19	94.12	84.21	15.00

**Table 3:** Feature importance from SelectFromModel based on random forest model. Features are ordered according to their importance score.

Order	Feature
1	Delay in delivering material
2	Prices fluctuation
3	Shortage of labourers
4	Unavailability of equipment
5	Construction cost underestimation
6	Delayed payment
7	Cash flow problem
8	High rate of interest
9	Increase in salaries
10	Change design
11	Errors and omissions in design
12	Inaccurate quantity take-off
13	Delays in issuing information
14	Delays in decisions making
15	Insufficient time for documents
16	Extension of time
17	Rework due to error in the execution
18	Accidents during construction
19	Delay in getting the ‘no objection certificate’

**Figure 4:**
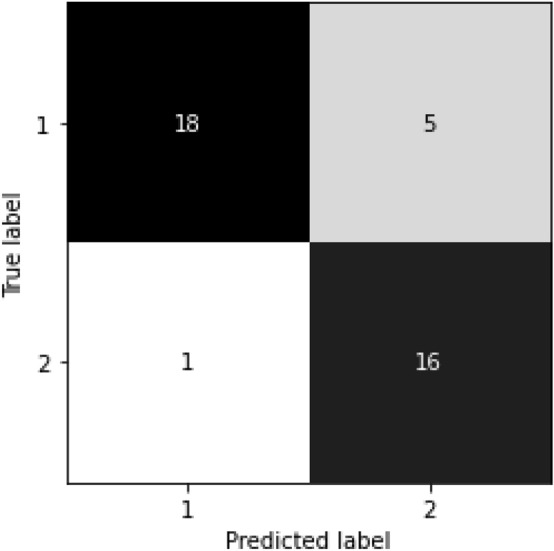
Confusion matrix results based on the random forest model with the SFM feature optimiser (1 for the rare class and 2 for the often class).

Further, Fig. 4 illustrates the confusion matrix when the random forest model with the SFM feature optimiser was applied to the test data. There are 18 true-positive, five false-negative, one false-positive and 16 true-negative cases. Therefore, the accuracy for the test phase is (18 + 16)/(18 + 5 + 1 + 16) = 85.00%.

**Figure 5:**
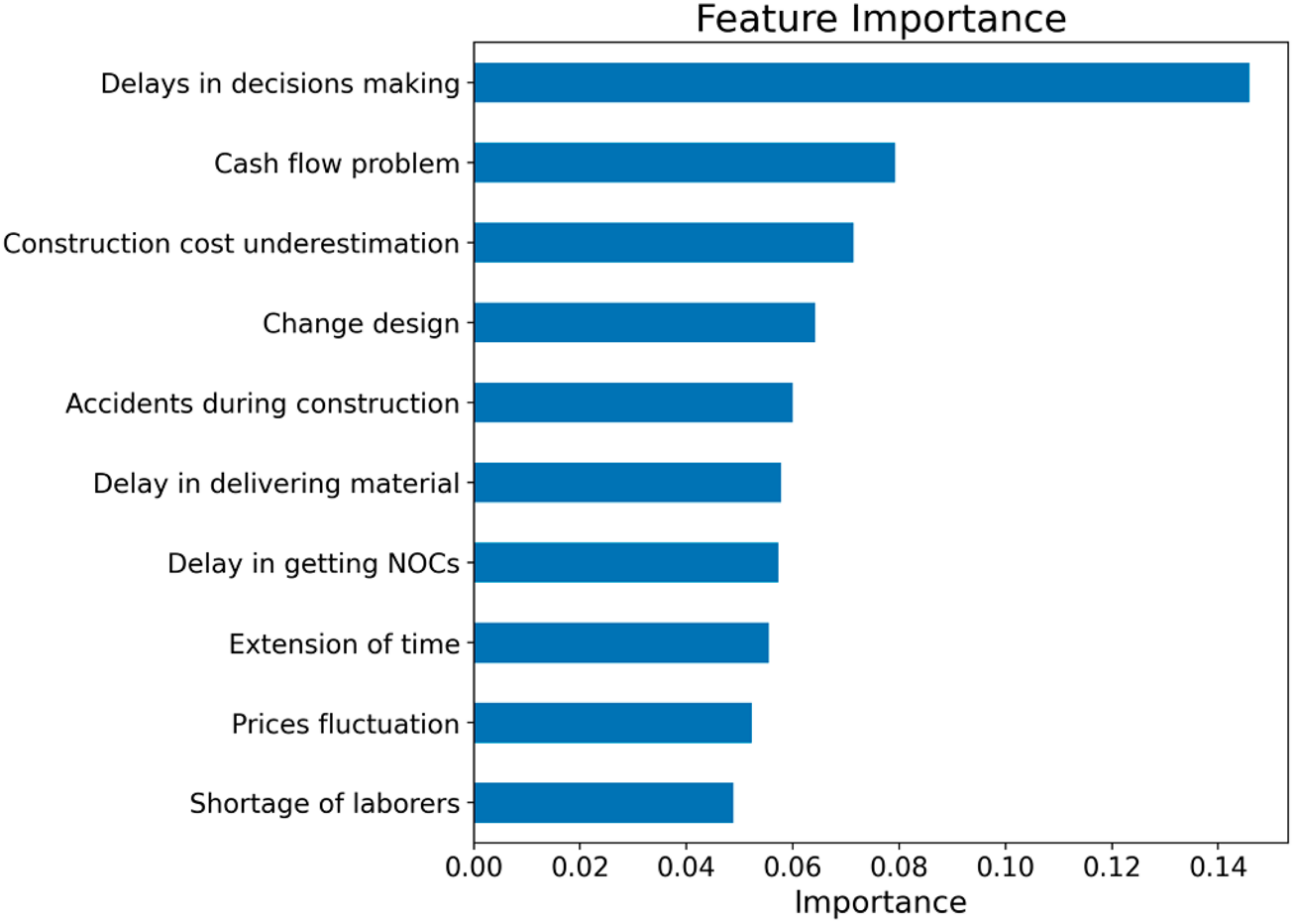
Feature importance (top-10 out of 19) based on the random forest model with the SFM feature optimiser.

Figure 5 illustrates the top-10 most important features or variables based on the random forest algorithm with the SFM optimiser. We used feature importance based on the mean decrease in impurity in identifying this list of important variables. Mean decrease in impurity computes each feature’s importance as the sum over the number of splits that include the feature in proportion to the number of samples it splits^71^. According to this figure, the *delays in decision marking* attribute contributed most to the classification performance of the random forest algorithm, followed by *cash flow problem* and *construction cost underestimation* attributes. The current construction project literature also highlighted these top-10 factors as significant contributors to project cost overrun. For example, using construction project data from Jordan, Al-Hazim et al.^72^ ranked 20 causes for cost overrun, including causes similar to these causes.

**Figure 6:**
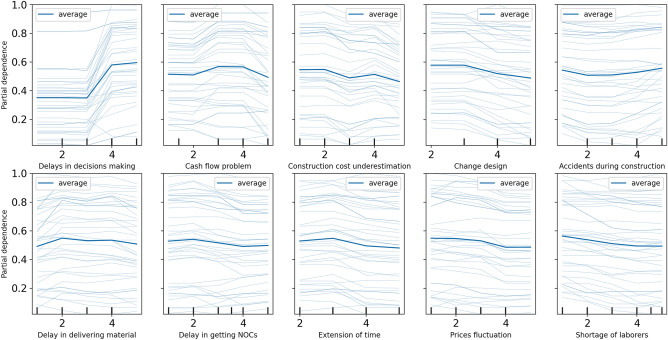
The result of the sensitivity analysis from the partial dependency plot tool for the ten most important features.

Further, we conduct a sensitivity analysis of the model’s ten most important features (from Fig. 5) to explore how a change in each feature affects the cost overrun. We utilise the partial dependence plot (PDP), which is a typical visualisation tool for non-parametric models ^73^, to display this analysis’s outcomes. A PDP can demonstrate whether the relation between the target and a feature is linear, monotonic, or more complicated. The result of the sensitivity analysis is presented in Fig. 6. For the ‘delays in decisions making’ attribute, the PDP shows that the probability is below 0.4 until the rating value is three and increases after. A higher value for this attribute indicates a higher risk of cost overrun. On the other hand, there are no significant differences can be seen in the remaining nine features if the value changes.

### Summary of the case study

We illustrated an application of the proposed machine learning-based research framework in classifying construction projects. RF showed the highest accuracy in predicting the test dataset. For a new data instance with information for its 19 features but has not had any information on its classification, RF can identify its class (*rare* or *often*) correctly with a probability of 85.00%. If more data is provided, in addition to the 139 instances of the case study, to the machine learning algorithms, then their accuracy and efficiency in making project classification will improve with subsequent training. For example, if we provide 100 more data instances, these algorithms will have an additional 50 instances for training with a 70:30 split. This continuous improvement facility put the machine learning algorithms in a superior position over other traditional methods. In the current literature, some studies explore the factors contributing to project delay or cost overrun. In most cases, they applied factor analysis or other related statistical methods for research data analysis^72,74,75^. In addition to identifying important attributes, the proposed machine learning-based framework identified the ranking of factors and how eliminating less important factors affects the prediction accuracy when applied to this case study.

We shared the Python software developed to implement the four machine learning algorithms considered in this case study using GitHub^76^, a software hosting internet site. user-friendly version of this software can be accessed at https://share.streamlit.io/haohuilu/pa/main/app.py. The accuracy findings from this link could be slightly different from one run to another due to the hyperparameter settings of the corresponding machine learning algorithms.

## Discussion

Due to their robust prediction ability, machine learning methods have already gained wide acceptability across a wide range of research domains. On the other side, EVM is the most commonly used method in project analytics due to its simplicity and ease of interpretability^77^. Essential research efforts have been made to improve its generalisability over time. For example, Naeni et al.^34^ developed a fuzzy approach for earned value analysis to make it suitable to analyse project scenarios with ambiguous or linguistic outcomes. Acebes^78^ integrated Monte Carlo simulation with EVM for project monitoring and control for a similar purpose. Another prominent method frequently used in project analytics is the time series analysis, which is compelling for the longitudinal prediction of project time and cost^30^. Apparently, as evident in the present current literature, not much effort has been made to bring machine learning into project analytics for addressing project management research problems. This research made a significant attempt to contribute to filling up this gap.

Our proposed data-driven framework only includes the fundamental model development and application process components for machine learning algorithms. It does not have a few advanced-level machine learning methods. This study intentionally did not consider them for the proposed model since they are required only in particular designs of machine learning analysis. For example, the framework does not contain any methods or tools to handle the *data imbalance* issue. *Data imbalance* refers to a situation when the research dataset has an uneven distribution of the target class^79^. For example, a binary target variable will cause a *data imbalance* issue if one of its class labels has a very high number of observations compared with the other class. Commonly used techniques to address this issue are undersampling and oversampling. The undersampling technique decreases the size of the majority class. On the other hand, the oversampling technique randomly duplicates the minority class until the class distribution becomes balanced^79^. The class distribution of the case study did not produce any data imbalance issues.

This study considered only six fundamental machine learning algorithms for the case study, although many other such algorithms are available in the literature. For example, it did not consider the extreme gradient boosting (XGBoost) algorithm. XGBoost is based on the decision tree algorithm, similar to the random forest algorithm^80^. It has become dominant in applied machine learning due to its performance and speed. Naïve Bayes and convolutional neural networks are other popular machine learning algorithms that were not considered when applying the proposed framework to the case study. In addition to the three feature selection methods, multi-view can be adopted when applying the proposed framework to the case study. Multi-view learning is another direction in machine learning that considers learning with multiple views of the existing data with the aim to improve predictive performance^81,82^. Similarly, although we considered five performance measures, there are other potential candidates. One such example is the area under the receiver operating curve, which is the ability of the underlying classifier to distinguish between classes^48^. We leave them as a potential application scope while applying our proposed framework in any other project contexts in future studies.

Although this study only used one case study for illustration, our proposed research framework can be used in other project analytics contexts. In such an application context, the underlying research goal should be to predict the outcome classes and find attributes playing a significant role in making correct predictions. For example, by considering two types of projects based on the time required to accomplish (e.g., *on-time* and *delayed*), the proposed framework can develop machine learning models that can predict the class of a new data instance and find out attributes contributing mainly to this prediction performance. This framework can also be used at any stage of the project. For example, the framework’s results allow project stakeholders to screen projects for excessive cost overruns and forecast budget loss at bidding and before contracts are signed. In addition, various factors that contribute to project cost overruns can be figured out at an earlier stage. These elements emerge at each stage of a project’s life cycle. The framework’s feature importance helps project managers locate the critical contributor to cost overrun.

This study has made an important contribution to the current project analytics literature by considering the applications of machine learning within project management. Project management is often thought of as being very fluid in nature, and because of this, applications of machine learning are often more difficult. Further, existing implementations have largely been limited to safety monitoring, risk prediction and cost estimation. Through the evaluation of machine learning applications, this study further demonstrates the uses for which algorithms can be used to consider and model the relationship between project attributes and cost overrun frequency.

## Conclusion

The applications of machine learning in project analytics are still undergoing constant development. Within construction projects, its applications have been largely limited and focused on profitability or the design of structures themselves. In this regard, our study made a substantial effort by proposing a machine learning-based framework to address research problems related to project analytics. We also illustrated an example of this framework’s application in the context of construction project management.

Like any other research, this study also has a few limitations that could provide scopes for future research. First, the framework does not include a few advanced machine learning techniques, such as data imbalance issues and kernel density estimation. Second, we considered only one case study to illustrate the application of the proposed framework. Illustrations of this framework using case studies from different project contexts would confirm its robust application. Finally, this study did not consider all machine learning models and performance measures available in the literature for the case study. For example, we did not consider the Naïve Bayes model and precision measure in applying the proposed research framework for the case study.

## Supplementary Information

Supplementary Information.

## Data Availability

This study obtained research data from publicly available online repositories. We mentioned their sources using proper citations. Here is the link to the data https://www.kaggle.com/datasets/amansaxena/survey-on-road-construction-delay.
