# Supplementary material for: Machine learning in project analytics: a data-driven framework and case study
Source: Sci Rep. 2022 Sep 9;12:15252. doi: 10.1038/s41598-022-19728-x (PMC9463441; doi:10.1038/s41598-022-19728-x)
Supplement: Supplementary file 1 — Supplementary Information. [file 41598_2022_19728_MOESM1_ESM.docx]

**Machine Learning in Project Analytics: A Data-Driven Framework and Case Study**

Model development

Final evaluation

Supplementary Figure 1: *k*-fold cross-validation for *k*=5. The shaded folds are the validation fold in each iteration. The final trained model is the aggregation from these five iterations. The test data is used after developing training models for evaluation purposes.


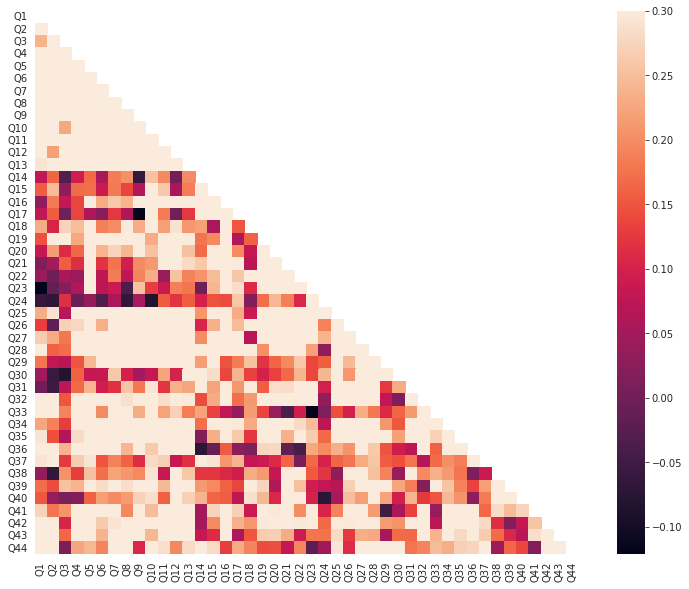


Supplementary Figure 2: The correlation matrix among the 44 features of the case study


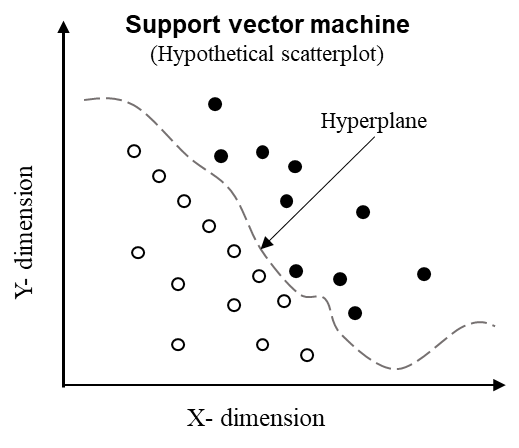

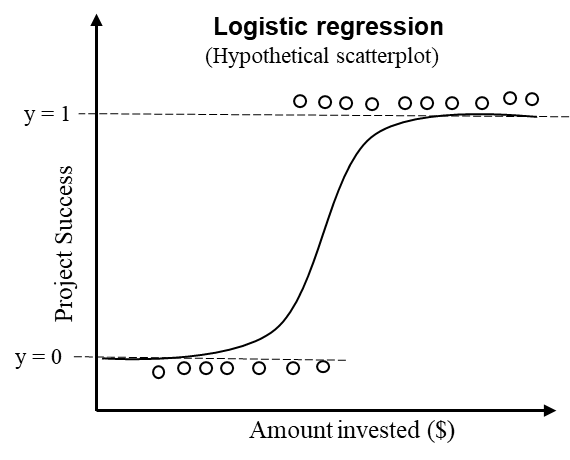

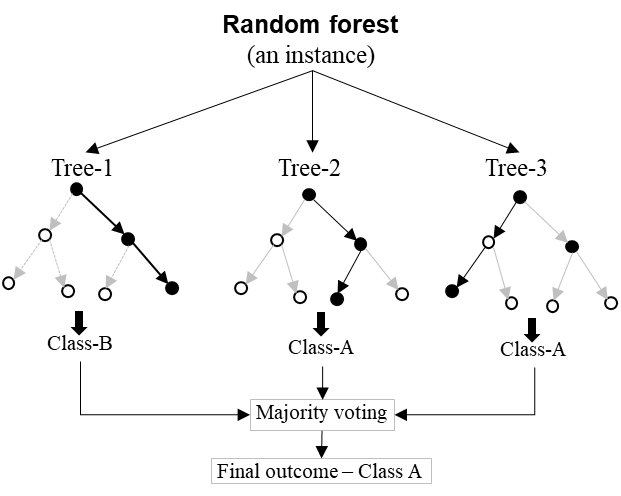

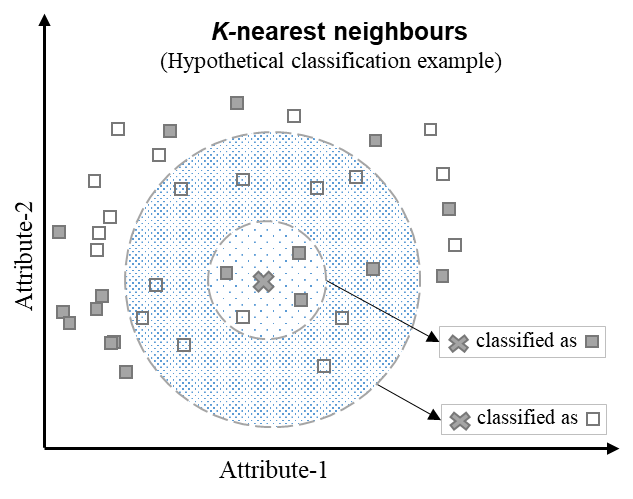


**(a)**

**(c)**

**(b)**

**(d)**


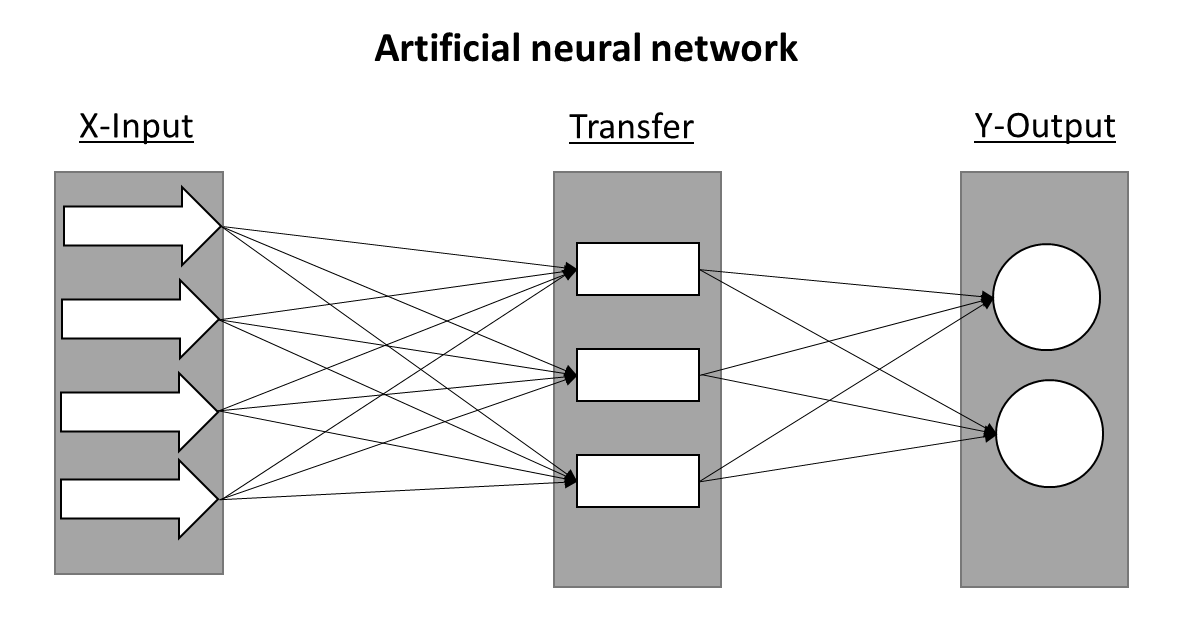

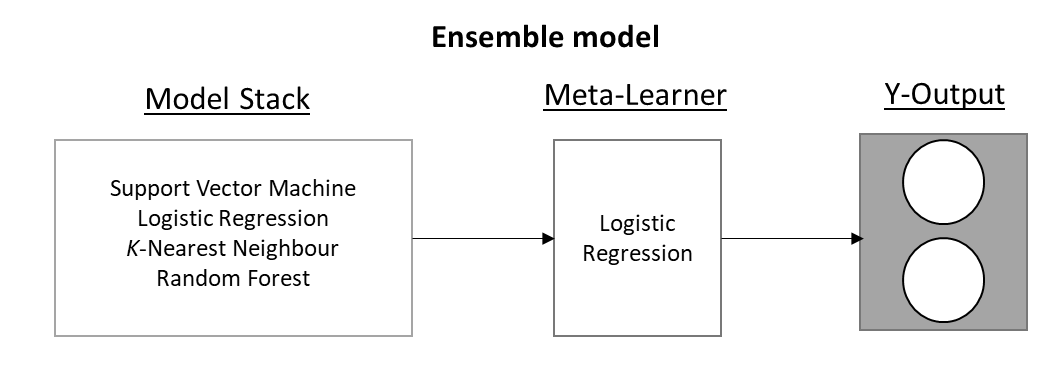


**(e)**

**(f)**

Supplementary Figure 3: The six machine learning algorithms considered for the case study.

Supplementary Table 1: The hyperparameter settings for the six algorithms considered in this study

| **Model** | **Parameters** |
| --- | --- |
| Logistic regression | *penalty = l2, C = 1, maximum iterations = 100, solver = newton-cg* |
| Support vector machine | *C = 10, gamma = scale, kernel = rbf* |
| k-nearest neighbours | *Metric: Euclidean, n_neighbours = 3, weights = distance* |
| Random forest | *criterion = gini, max feature = sqrt, number of estimators = 100,* |
| Stacking | *Base models: default LR, SVM, KNN and RF, meta learner: LR* |
| Artificial neural network | *Number of layers = 3, number of neurons = 64, activation function: ReLU for hidden layers and sigmoid for output layer, learning rate = 0.01, loss function = binary cross entropy, optimiser = Adam* |
